# *In situ* Growth of Cu_2_O/CuO Nanosheets on Cu Coating Carbon Cloths as a Binder-Free Electrode for Asymmetric Supercapacitors

**DOI:** 10.3389/fchem.2019.00420

**Published:** 2019-06-06

**Authors:** Lina Xu, Jiao Li, Haibin Sun, Xue Guo, Jiakun Xu, Hua Zhang, Xiaojiao Zhang

**Affiliations:** ^1^School of Materials Science and Engineering, Shandong University of Technology, Zibo, China; ^2^Yellow Sea Fisheries Research Institute, Chinese Academy of Fishery Sciences, Qingdao, China

**Keywords:** copper oxide, nanostructures, electrode, carbon cloth, asymmetric supercapacitor

## Abstract

Cu_2_O/CuO nanosheets *in-situ* grown on Cu-Carbon cloths (Cu-CCs), namely Cu_2_O/CuO@Cu-CCs, are constructed by a simple strategy with electroless copper plating, chemical etching, and thermal dehydration. The as-prepared material is directly used as binder-free electrodes for supercapacitors (SCs). CCs coated with Cu, as the current collector, can effectively promote the charge collection and electron transfer, while the hierarchical Cu_2_O/CuO nanosheets provide massive active sites for fast faradic reactions. The composite electrode exhibits high specific capacitance [1.71 F cm^−2^, equivalent to 835.2 F g^−1^, at the current density of 10 mA cm^−2^ (3.57 A g^−1^)]. The asymmetric supercapacitor device using Cu_2_O/CuO@Cu-CCs as the positive electrode and activated carbon as the negative electrode, achieves a superior energy density up to 60.26 Wh kg^−1^ at a power density of 299.73 W kg^−1^ and an excellent long-term cycling stability (9.65% loss of its initial capacitance after 5,000 cycles). The excellent electrochemical performance is mainly ascribed to the unique hierarchical structure of Cu_2_O/CuO@Cu-CCs, making it attractive as a potential electrode material for high performance SCs.

## Introduction

Supercapacitors (SCs), one of the most promising energy storage devices, have received extensive attention owning to their high power density, fast charge/discharge speed, long cycling life span, and low-cost (Lu et al., 2014; Xiong et al., 2015; Sami et al., 2017; Dai et al., 2018). According to the reaction mechanisms, SCs can be classified into electrical double layer capacitors (EDLCs) and pseudocapacitors (PCs) (Wei et al., 2012). For EDLCs, the charges are stored electrostatically at the electrode/electrolyte interface while typically taking carbon materials as active materials (Surendran et al., 2018). For PCs, the energy is stored within the electrode through the faradic redox reaction while taking transition metal oxides/hydroxides and conducting polymers as the electrode materials, thus the PCs provide much higher energy density and specific capacitance than EDLCs. Nevertheless, there are many of problems scarcely understood which attract large numbers of investigator devote oneself to resolve, such as inadequate energy density and capacitance, poor electrochemical stability for practical applications.

In recent years, various transition metal oxides/hydroxides, such as RuO_2_ (Wang et al., 2014), NiO (Ouyang et al., 2019), Ni(OH)_2_ (Kim et al., 2017), MnO_2_ (Huang et al., 2015), Co_3_O_4_ (Liu T. et al., 2018), Co(OH)_2_ (Yang et al., 2018), V_2_O_5_ (Foo et al., 2014), CuO (Bu and Huang, 2017; Li et al., 2017; Liu Y. et al., 2018), Cu_2_O (Zhang W. et al., 2016; Ji et al., 2017), have been applied to achieve excellent capacitive performance for PCs. Among these materials, CuO, Cu_2_O, or Cu_2_O/CuO nanostructures with different configurations including nanoneedle, nanoflowers, nanowires (Dong et al., 2014; Wang et al., 2015; Chen et al., 2016; Xu et al., 2016; Yang et al., 2016), are attracting considerable interest due to their environmental friendliness, numerous reserve, low-cost, chemical stability, and excellent electrochemical properties [theoretical capacitance of CuO up to 1,800 F g^−1^ (Liu Y. et al., 2018) and Cu_2_O is up to 2,247 F g^−1^ (Wu et al., 2017)]. However, most of metal oxides/hydroxides were poor in electrical conductivity, making it difficult to achieve high specific capacitance (Xu et al., 2016). To resolve this issue, oxides/hydroxides are typically mixed with ancillary carbon black or binder and then bonded to current collector, but leading to a significant decrease of the overall specific capacitance (Yuan et al., 2017). An effective approach is that, nanostructured electrode materials directly grow on current collectors, forming binder-free electrodes, thus achieving higher energy density (Dong et al., 2014).

Carbon material containing carbon nanotube, graphene and carbon fiber is one kind of the preferred current collectors due to their excellent electrical conductivity and electrochemical stability (Prasad et al., 2011; Moosavifard et al., 2014; Bu and Huang, 2017). Among various carbon materials, carbon cloths (CCs) with low-cost, chemical stability and desirable conductivity, are regarded as novel carbonaceous materials, which are consist of numerous uniform carbon fibers with three-dimensional (3D) structure (Guo et al., 2014; Zhang Y. et al., 2016). The 3D network structure is conducive to shorten the diffusion pathway of ions and accelerate the flow of ions during the electrochemical process. Numerous electrode materials of PCs taking CCs as current collectors have been developed, such as NiCo-LDH@NiOOH (622 F g^−1^ at 1 A g^−1^) (Liang et al., 2018), MnNiCoO_4_@MnO_2_ (1931 F g^−1^ at 0.8 A g^−1^) (Saray and Hosseini, 2016), MnO_2_ nanosheet arrays (2.16 F cm^−2^, at 5 mA cm^−2^) (Guo et al., 2014). Currently, copper oxide and its composite materials are mainly grown on copper foam and copper foil (Zhang et al., 2015; Singh and Sarkar, 2017), and the combination of CuO or Cu_2_O with CCs is also in the developing situation. For example, Xu et al. (2016) fabricated CuO nanoflower arrays on CCs, the energy density and power density are 10.05 Wh kg^−1^ and 1,798.5 W kg^−1^, respectively. Wan et al. (2017) developed forest-like cuprous oxide/copper with the energy density of 24.0 Wh kg^−1^ at 0.625 kW kg^−1^. However, it is still challenging to evolve the commercially viable Cu oxides/hydroxides with high energy/power density, specific capacitance, and excellent cycling stability (Dong et al., 2014). Therefore, it will be worthy to make a thorough research on CuO or Cu2O electrodes grown on CCs.

In order to improve the kinetics and electrochemical performance of electrodes, two typical methods are usually employed. One straightforward approach is to increase the specific surface area of electrodes to provide more active sites for faradaic redox reaction (Daoping et al., 2014). The other method is to improve the conductivity of electrode material to accelerate electron conduction (Lu et al., 2013). Herein, we firstly synthesized Cu_2_O/CuO nanosheets directly grown on CCs which is coated with Cu film by a simple strategy with electroless copper plating, chemical etching and thermal dehydration. The uniform Cu film on carbon microfiber cloth has a strong binding force. In addition, Cu_2_O/CuO nanosheets *in situ* grown on CCs provide sufficient active sites for charge/discharge electronic, which is important for energy storage of supercapacitor. Finally, it is worth mentioning that there are still Cu films between CCs and Cu_2_O/CuO nanosheets after chemical etching, which is important for promoting electronic conduction.

## Experimental

### Materials Synthesis

CCs (WOS1002) were purchased from CeTech. (NH_4_)_2_S_2_O_8_ (Tianjin Huachen Company) and all other reagents (from Aladdin) were of analytical grade without further treatment. In a typical electroless copper plating process, CCs, cut into squares (25 × 25 mm), were firstly heated to 400°C at a heating rate of 10°C min^−1^ and hold for 30 min in muffle furnace under air atmosphere to remove a part of impurities. And then, the CCs were immersed into concentrated nitric acid to make the surface rough, followed by the sensitization and activation treatment. Stannous chloride/hydrochloric acid and silver nitrate/ammonium hydroxide solutions were used as the sensitizer and activator, respectively (Yuan et al., 2017). The composition of the sensitizing and activating solution are shown in Supplementary Tables 1, 2. The sensitization and activator treatment adsorbs a layer of active silver particles on the surface of the carbon cloth as active metal particles, and copper ions were first reduced on the active metal particles, so that the reduction reaction of copper proceeds on the surface of the carbon cloth. Catalyzed CCs with a number of active sites were obtained after in NaOH (10%) for 3 min. Subsequently, the catalyzed CCs were immersed into plating solutions and stirred at a rotating speed of 200 r min^−1^ for 60 min at 25°C, during which Cu films were coated on CCs, thus obtaining Cu-CCs samples. The amount of copper retained is about 0.009 g cm^−2^ on the carbon cloth. The composition of the electroless copper plating solution is shown in Supplementary Table 3. Formaldehyde is used as a reducing agent, and the main chemical reactions in electroless copper plating solutions are as follows:

$$\begin{array}{l} \text{C}{\text{u}}^{2+}+2\text{HCHO}+4\text{O}{\text{H}}^{-}\to \text{Cu}+2\text{HCO}{\text{O}}^{-}+{\text{H}}_{2}\text{↑}+{\text{H}}_{2}\text{O}\\ 2\text{C}{\text{u}}^{2+}+\text{HCHO}+5\text{O}{\text{H}}^{-}\to \text{C}{\text{u}}_{2}\text{O}+\text{HCO}{\text{O}}^{-}+\;3{\text{H}}_{2}\text{O}\\ \text{C}{\text{u}}_{2}\text{O}+2\text{HCHO}+2\text{O}{\text{H}}^{-}\to 2\text{Cu}+2\text{HCO}{\text{O}}^{-}+{\text{H}}_{2}\text{↑}+{\text{H}}_{2}\text{O} \end{array}$$

In the chemical etching process, the Cu-CCs were dipped into 100 mL mixed solutions with 2.5 mol L^−1^ NaOH and 0.1 mol L^−1^ (NH_4_)_2_S_2_O_8_ at 25°C for a while, Cu(OH)_2_ arrays were *in situ* grown on Cu-CCs. After being washed, Cu(OH)_2_ arrays were decomposed into Cu_2_O/CuO arrays through a thermal dehydration at 120°C in air for 3 h, thus obtaining Cu_2_O/CuO@Cu-CCs electrodes.

### Materials Characterization and Electrochemical Measurements

The phase compositions of the products were identified by X-ray diffraction analysis (XRD, Rigaku-Dmax 2500 diffractometer). The microstructure and morphology were observed by scanning electron microscopy (SEM, HITACHI S4800) and high-resolution transmission electron microscopy (HRTEM, Tecnai G2 F20 STWIN, FEI, USA). X-ray photoelectron spectroscopy (XPS, Kratos Axis Ultra DLD, Britain) was performed using Mg Ka as the exciting source.

Cyclic voltammetry (CV) and galvanostatic charge-discharge (GCD) tests of Cu_2_O/CuO@Cu-CCs electrodes were tested on a CHI 660E electrochemical workstation (Shanghai Chenhua Instrument Company, China) in a three-electrode electrochemical cell using a 6 M KOH aqueous solution as the electrolyte at room temperature. The Cu_2_O/CuO@Cu-CCs electrodes were used as the working electrode, while a platinum wire and an Ag/AgCl electrode as the counter and reference electrode, respectively. Electrochemical impedance spectroscopy (EIS) tests were performed in the frequency ranging from 106 to 0.01 Hz. The specific capacitances were calculated from the discharge part of the GCD curves using the following equation.

(1) $$\begin{array}{l} \text{C}=\left(\text{I}\int \text{Vdt}\right)/\left(\text{S}{\text{V}}^{2}\right) \end{array}$$

where *C* represents the specific capacitance (F cm^−2^), I represents the discharge current (A), Δ*t* is the total discharging time (s), *S* is the area of the sample (cm^2^), and Δ*V* is the potential change (V) within the discharge time Δ*t*.

### Fabrication and Electrochemical Measurements of Asymmetric Supercapacitor

Active carbon, acetylene black, and poly tetra fluoroethylene (PTFE) with a mass ratio of 80:10:10 were mixed with moderate amount of ethanol. The resulting mixture was brushed on carbon cloth and dried at 80°C for 10 h in a vacuum oven. Acetylene black and PTFE are acted as conductive agents and binders, respectively. The asymmetric supercapacitor (ASC) device was assembled by using Cu_2_O/CuO@Cu-CCs electrode (with a diameter of 1 cm) and active carbon electrode as the positive and negative electrode, respectively. The filter papers soaked with 6 M KOH solution were taken as separators. As a electrochemical property's asymmetric supercapacitor, the charge stored between the two electrodes should keep the balance relationship (q^+^ = q^−^), which could be calculated by equation (Liu Y. et al., 2018).

(2) $$\begin{array}{l} \text{q}=\text{C}•\text{m}•\text{ΔV} \end{array}$$

where *C* represents the specific capacitance (F g^−1^), m is the mass of active materials on both electrodes (g), Δ*V* is the potential window (V). Therefore, the mass ratio of electroactive material between the two electrodes could be calculated by equation (Li et al., 2019).

(3) $$\begin{array}{l} {\text{m}}^{+}/{\text{m}}^{-}={\text{C}}^{-}\text{Δ}{\text{V}}^{-}/{\text{C}}^{+}\text{Δ}{\text{V}}^{+} \end{array}$$

where *C*^−^ (F g^−1^) and Δ*V*^−^ (V) are the specific capacitance and the voltage range of scanning segment of the AC electrode, respectively. *C*^+^ (F g^−1^) and Δ*V*^+^ (V) are the specific capacitance and the voltage range of scanning segment of the Cu_2_O/CuO@Cu-CCs electrode. The specific capacitance, energy density and power density of the ACS device were calculated using the following equations (Ensafi et al., 2018; Liu Y. et al., 2018).

(4) $$\begin{array}{l} \text{Cs}=\left(2\text{I}\int \text{Vdt}\right)/\left(\text{m}{\text{V}}^{2}\right) \end{array}$$

(5) $$\begin{array}{l} \text{E}=1/2\text{CΔ}{\text{V}}^{2} \end{array}$$

(6) $$\begin{array}{l} \text{P}=\text{E}/\text{Δt} \end{array}$$

where *Cs* represents the specific capacitance (F g^−1^), *I* is the discharge current (A), Δ*V* is the potential window (V), Δt is the discharge time (s), m is the mass of active materials on both electrodes (g), *E* and *P* correspond to the energy density (Wh kg^−1^) and power density (W kg^−1^), respectively (Guan et al., 2017).

## Results and Discussion

**Figure 1:**
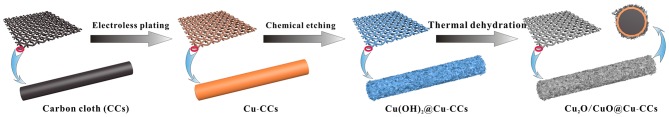
Schematic illustration of the growth process of Cu_2_O/CuO nanosheets on Cu coating carbon cloth to prepare Cu_2_O/CuO@Cu-CCs electrodes.

The schematic illustration of the growth process of Cu_2_O/CuO@Cu-CCs electrodes is shown in Figure 1. Firstly, the Cu film is uniformly coated on the CCs through electroless copper plating, forming Cu-CCs samples. Subsequently, Cu(OH)_2_ nanosheet arrays are *in situ* grown on Cu film by alkaline oxidative enchanting in NaOH and (NH_4_)_2_S_2_O_8_ solution, during which the oxidative S_2_${\text{O}}_{8}^{2-}$ is attached on the surface of Cu-CCs, and partial CuO are oxidized to Cu^2+^(Chen et al., 2016). With the reaction of Cu^2+^ and OH^−^, Cu(OH)_2_ nanosheet arrays are formed and then are decomposed into Cu_2_O/CuO nanosheets by thermal dehydration, thus obtaining Cu_2_O/CuO@Cu-CCs electrodes

**Figure 2:**
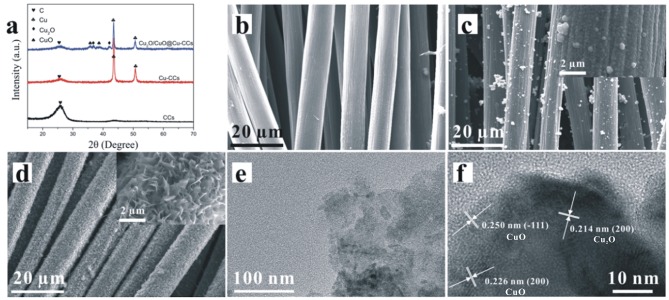
**(a)** XRD patterns of bare CCs, Cu-CCs, and Cu_2_O/CuO@Cu-CCs samples. SEM images of bare **(b)** CCs, **(c)** Cu-CCs, and **(d)** Cu_2_O/CuO@Cu-CCs samples. **(e)** TEM image and **(f)** HRTEM image of Cu_2_O/CuO nanosheets.

XRD patterns of CCs, Cu-CCs and Cu_2_O/CuO@Cu-CCs are shown in Figure 2a. As can be seen by comparing peaks of CCs and Cu-CCs, the Cu films on CCs leads to the decrease of the characteristic peaks of carbon fiber at 2θ = 26.4°. For Cu-CCs samples, there are two strong diffraction peaks at 2θ = 43.5 and 50.6°, corresponding to the (111) and (200) planes of the metallic copper (JCPDS no. 04-0836), respectively (Chen et al., 2016). After the heat treatment at 120°C, the sample exhibits four peaks at 2θ of 35.6, 36.4, 39.1, 42.3°, in which 2θ = 35.6 and 39.1° correspond to (−111) and (200) planes of the CuO substrates (JCPDS no. 48-1548), while the else two peaks (2θ = 36.4 and 42.3°) are attributed to the (111) and (200) reflections of Cu_2_O (JCPDS no. 05-0667). It is worthy to note that Cu and CCs peaks are still observed, therefore, the composition is confirmed to be Cu_2_O/CuO@Cu-CCs.

Shown in Figure 2b is the SEM image of bare CCs, it can be observed that the surface of the carbon fibers is smooth and the diameter is around 8–10 μm. In Figure 2c, the carbon fibers are uniformly coated with copper films. After being etched in alkaline solution, the morphologies of Cu_2_O/CuO nanosheets vary with the change of etching time (Supplementary Figure 1). With an etching time of 25 min, the sample exhibits a highly porous cross-linked structure with abundant thin Cu_2_O/CuO nanosheets (Figure 2d). The lamellar nanosheets can effectively increase the number of active sites, which may be beneficial for promoting charge transfer and redox reaction (Liu Y. et al., 2018). As shown in Figure 2e, the porous Cu_2_O/CuO nanosheets are ultra-thin, which may enlarge the specific surface area to accelerate the intercalation and de-intercalation of ions (Chen et al., 2016). In addition, the HRTEM image in Figure 2f shows that the measured interplanar spacing of 0.250 and 0.226 nm for the well- defined lattice fringes are consistent well with the (−111) and (200) plane of CuO (JCPDS no. 48-1548), and there is a part of interplanar distances calculated to be 0.214 nm, which can be directed as the (200) plane of Cu_2_O (JCPDS no. 05-0667)

**Figure 3:**
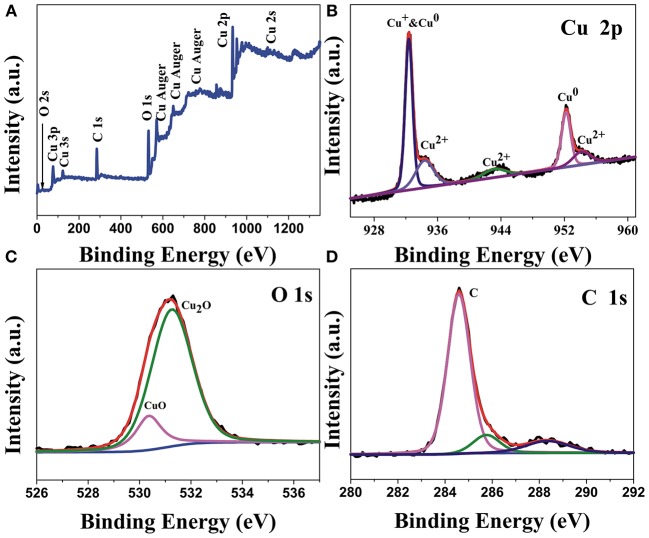
**(A)** XPS survey spectrum of Cu_2_O/CuO@Cu-CCs samples and high relation XPS spectra for **(B)** Cu 2p, **(C)** O 1s, **(D)** C 1s.

The XPS spectrums of the surface atomic composition and chemical state of the Cu_2_O/CuO@Cu-CCs samples are obtained by Gaussian curve-fitting. As illustrated in Figure 3A, the complete spectrum indicates the existence of C, Cu, and O elements in the sample. As shown in Figure 3B, there are two sharp peaks located at 932.43 and 952.5 eV, which are correspond to Cu 2p3/2 and Cu 2p1/2, respectively, illustrating the coexistence of Cu^+^ and Cu^0^ species (Wan et al., 2017). At the same time, there are three satellite peaks with binding energies of 934.2, 943.1, and 953.9 eV indicated the existence of CuO in the samples. Therefore, it can be concluded that the copper is mainly Cu^2+^, Cu^+^, and Cu^0^ (Liu Y. et al., 2018). The existence of Cu^0^ can be beneficial for improving the electronic conduction of electrodes. As shown in Figure 3C, the O 1s XPS spectrum can be deconvoluted into two peaks, one is the peak at 530.3 eV, which represents the oxygen in Cu_2_O lattice. Another is the high intensity peak at 531.1 eV, which is attributed to the CuO. This result further confirms the coexistence of CuO and Cu_2_O (Singh and Sarkar, 2017). In the Figure 3D, C 1s spectrum shows a high intensity peak at 284.5 eV, demonstrating that the intensity of C-C functional group peak is notably strong, further illustrating carbon fiber is stable in Cu_2_O/CuO@Cu-CCs.

**Figure 4:**
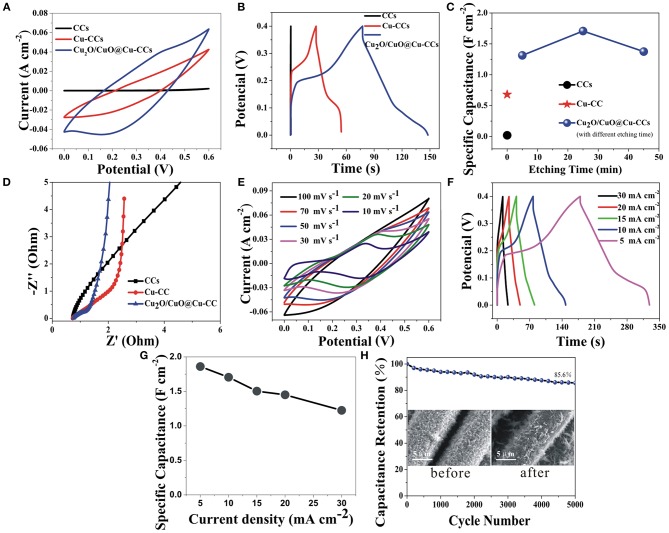
CV curves at a scan rate of 30 mV s^−1^ **(A)**, GCD curves at a current density of 10 mA cm^−2^ **(B)**, the capacitance retention at 10 mA cm^−2^ **(C)**, and Nyquist plot **(D)** of CCs, Cu-CCs, and Cu_2_O/CuO@Cu-CCs electrodes in a 3-electrode cell in 6 M KOH aqueous solution. CV curves at various scan rates **(E)**, GCD curves under different current densities **(F)**, specific capacitances under different current densities **(G)**, and the cycling performance at a current density of 5 mA cm^−2^ (the inset shows the SEM images before and after 5,000 cycles) **(H)** of Cu_2_O/CuO@Cu-CCs electrode.

The CV curves of CCs, Cu-CCs and Cu_2_O/CuO@Cu-CCs at a scan rate of 30 mV s^−1^ are shown in Figure 4A. It is obvious that strong pair of anodic and cathodic peaks is clearly visible for Cu_2_O/CuO@Cu-CCs and Cu-CCs samples, mainly due to the Faradaic redox reaction (Dong et al., 2014). For Cu-CCs, copper ions mainly derive from the reaction of Cu and KOH electrolyte solution during electrochemical measurement. The pronounced pseudocapacitive characteristic of Cu_2_O/CuO@Cu-CCs is mainly attributed to the porous cross-linked Cu_2_O/CuO nanosheets while the contribution of capacitance for CCs can be negligible. Figure 4B shows the GCD curves of different electrodes at a constant current density of 10 mA cm^−2^. The non-linear behavior of GCD curves further verifies that the main sources for charge storage originate from Faradaic reactions. The Cu_2_O/CuO@Cu-CCs electrode discussed above is the sample etched for 25 min (CV and GCD curves of other samples are shown in Supplementary Figures 2A,B), and this sample shows the best pseudocapacitive characteristic with a specific capacitance of 1.71 F cm^−2^ (835.2 F g^−1^) at 10 mA cm^−2^ (3.57 A g^−1^) (Figure 4C), which is outperform the previously published values of Cu_2_O/CuO-based electrodes (1.674 F cm^−2^, equivalent to 594.27 F g^−1^, at 2 mA cm^−2^; 839.9 Fg^−1^, at 1 mVs^−1^; 357 F g^−1^, at 10 A g^−1^) and more exhaustive data were displayed in Supplementary Table 4. The EIS analysis was studied to further clarify the electrochemical behaviors of different electrodes. The Nyquist diagrams are shown in Figure 4D, which consist of an approximate semicircle in the high-frequency region and a line in the low-frequency region. All real-axis intercepts are as low as approximately 0.5 Ω, illustrating all the samples have excellent electronic conduction due to the CCs and Cu-CCs current collectors. The depressed semicircle at the high frequency region corresponds to charge transfer resistance (Rct) caused by Faradaic reactions (Ensafi et al., 2018). The Cu_2_O/CuO@Cu-CCs electrode has the smallest semicircle, illustrating an enhanced charge transfer. Also, the straight line in low-frequency region can be ascribed to Warburg impedance related to the fast charge diffusion in the electrolyte (Ensafi et al., 2018).

The electrochemical performances of Cu_2_O/CuO@Cu-CCs at various scan rates and current densities (Figures 4E,F) demonstrate a perfect reversibility during the charge-discharge process. Clearly, the slope of GCD curves decline suddenly at 0.18–0.25 V in charge part and the same as discharge part, corresponding the pseudocapacitance behavior in the CV scans, which is associated with the Faradaic redox reactions of Cu^2+^/Cu^+^ redox pairs related to OH^−^ as bellows (Guan et al., 2017; Sami et al., 2017).

$$\begin{array}{l} \text{CuO}+{\text{H}}_{2}\text{O}+2{\text{e}}^{-}↔\text{C}{\text{u}}_{2}\text{O}+\;2\text{O}{\text{H}}^{-}\\ \text{C}{\text{u}}_{2}\text{O}+{\text{H}}_{2}\text{O}+2\text{O}{\text{H}}^{-}↔2\text{Cu}{\left(\text{OH}\right)}_{2}+\;2{\text{e}}^{-}\\ \text{CuOH}+\text{O}{\text{H}}^{-}↔\text{Cu}{\left(\text{OH}\right)}_{2}+{\text{e}}^{-}\\ \text{CuOH}+\text{O}{\text{H}}^{-}↔\text{CuO}+{\text{H}}_{2}\text{O}+{\text{e}}^{-} \end{array}$$

Remarkable, with the current density increases from 5 to 30 mA cm^−2^, the GCD curves present a gradually decreased discharge time but tends to preserve similar shape (Figure 4F) and the electrode retains 68.5% of its capacitance (Figure 4G), suggesting an excellent rate capability. Furthermore, the Cu_2_O/CuO@Cu-CCs electrode delivers excellent cycling stability with only 14.4% loss in specific capacitance after 5,000 cycles at 5 mA cm^−2^ (Figure 4H), which can be explained by the stable structure of electrodes after cycling (the inset in Figure 4H).

**Figure 5:**
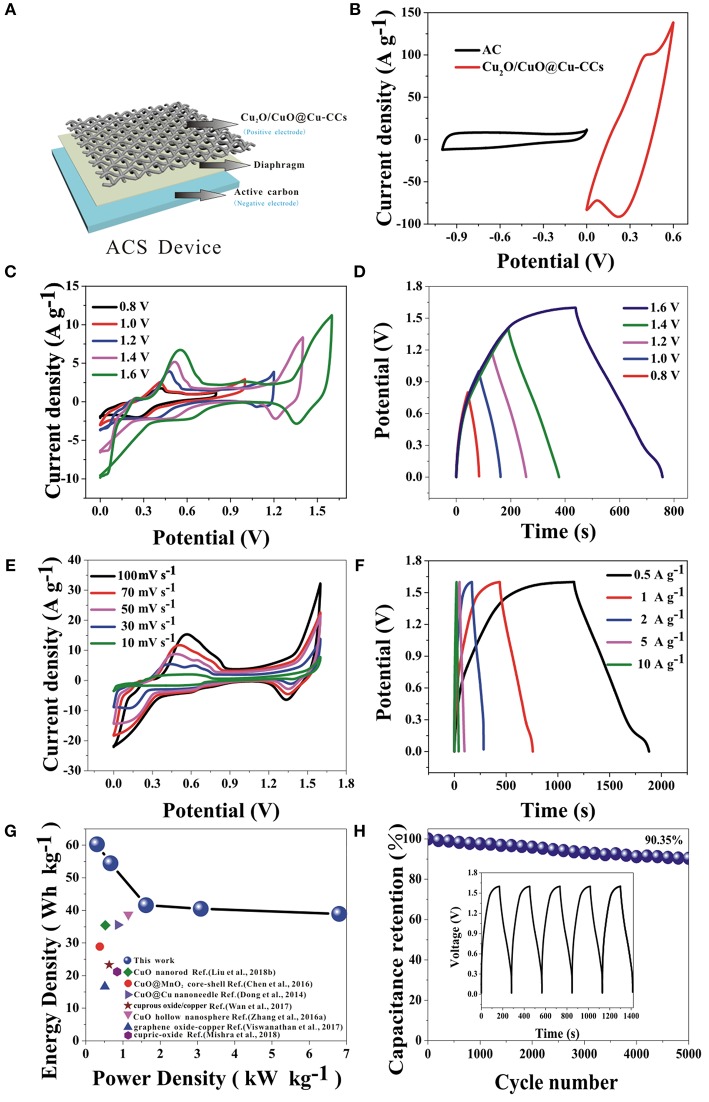
The schematic illustration **(A)** and comparative CV curves of Cu_2_O/CuO@Cu-CCs and AC electrodes at a scan rate of 30 mV s^−1^ in a three-electrode system **(B)**, electrochemical performances of the ASC device: **(C)** CV curves of at various potential windows at 30 mV s^−1^, **(D)** GCD curves at different potential windows at a current density of 1 A g^−1^, **(E)** CV curves at various scan rates, **(F)** GCD curves at increasing current densities, **(G)** the Ragone plot, and **(H)** the long-term cycling stability at a current density of 2 A g^−1^, the inset is the last 5 cycles of GCD curves.

For further exploring of the application, the electrochemical performances of the ASC device are investigated. As shown in Figure 5A, the device is sandwiched with the Cu_2_O/CuO@Cu-CCs positive electrode, active carbon negative electrode, and diaphragm separator soaked with 6 M KOH aqueous solution. Figure 5B shows the exactly complementary potential windows range of simple AC and Cu_2_O/CuO@Cu-CCs electrode, which suggest the high potential window of the ACS device. Furthermore, the calculated mass ratio of the electroactive materials of negative and positive electrodes according to Equation (3) is about 1:20. Figures 5C,**D** show the CV curves at a scan rate of 30 mV s^−1^ and GCD curves at a current density of 1 A g^−1^ with different potential windows, respectively. It is obvious that the shapes of the CV curves stay nearly same at different potential windows and the maximum potential window is extended to 1.6 V. The perfect symmetry and nearly unchanged shapes at different potential windows of GCD curves also contribute to the outstanding capacitive performance of this ASC device.

Figure 5E shows the CV curves of the ASC device at a scan rate ranging from 10 to 100 mV s^−1^. Apparently, the excellent synergy effect of the two electrodes leads to the high operation voltage of 1.6 V, which is three times as wide as the potential window of Cu_2_O/CuO@Cu-CCs electrode in the three-electrode system. Meanwhile, the curve shape retains the same at different scan rates. The GCD curves at current densities from 0.5 to 10 A g^−1^ are shown in Figure 5F. It is obvious that very low voltage drops are visible compared with three-electrode test even at high current densities. And the symmetrical shape indicates high reversibility of the device. Thus, the device shows excellent rate capability (Supplementary Figure 3). In addition, owing to the broad potential window and huge specific capacitance, the ASC device shows a high energy density of 60.26 Wh kg^−1^ at a power density of 299.73 W kg^−1^, higher than some other literatures (Figure 5G). In order to investigate the long-term cycling stability and durability of the device, we performed 5,000 continuous GCD cycles at a current density of 2 A g^−1^. The ASC device exhibits an excellent cycling stability with keeping 90.35% in its specific capacitance after 5,000 GCD cycles (Figure 5H). This kind of electrode material will be a promising electrode for further engineering all-solid-state high-performance supercapacitor due to its excellent capacitor performance and flexibility characteristic.

## Conclusions

In short, we constructed Cu_2_O/CuO@Cu-CCs electrodes by a simple process with electroless copper plating, chemical etching and thermal dehydration. The ASC device with Cu_2_O/CuO@Cu-CCs positive electrode and AC negative electrode showed high energy density of 60.26 Wh kg^−1^ at a power density of 299.73 W kg^−1^ using 6 M KOH aqueous solution as the electrolyte. Also, the ASC device express an excellent cycling stability with keeping 90.35% in its specific capacitance after 5,000 GCD cycles. Also, this kind of electrode material will be a promising electrode for further engineering all-solid-state high-performance supercapacitor.

## Data Availability

The raw data supporting the conclusions of this manuscript will be made available by the authors, without undue reservation, to any qualified researcher.

## Author Contributions

LX synthesized Cu_2_O/CuO@Cu-CCs samples and analyzed part of characterizations. JL was the supervisor of this research work. HS and XG helped with the data analysis. JX analyzed XPS measurements. HZ organized a part of the data. XZ supplemented a part of the experiment.

### Conflict of Interest Statement

The authors declare that the research was conducted in the absence of any commercial or financial relationships that could be construed as a potential conflict of interest.
