# Supplementary material for: In situ Growth of Cu2O/CuO Nanosheets on Cu Coating Carbon Cloths as a Binder-Free Electrode for Asymmetric Supercapacitors
Source: Front Chem. 2019 Jun 6;7:420. doi: 10.3389/fchem.2019.00420 (PMC6562678; doi:10.3389/fchem.2019.00420)
Supplement: Supplementary file 1 [file Data_Sheet_1.pdf]

frontiers

Supplementary Material

1 Supplementary Data

The mass of Cu_2O/CuO was calculated by means of the following steps. Firstly, the

mass of the completely clean and dried $Cu_2O/CuO@Cu$-CCs electrode, named m1, was weighed by electronic balance with an accuracy of 0.00001 g. Secondly, this electrode was immersed into a 1 M HCl solution about 5 min until no obvious colour change occurred. During this step, the Cu_2O/CuO nanosheets was removed. After washing with deionized water for several times, the remaining Cu-CCs were put in a vacuum oven at 60 ℃ for 4h. The mass of the remaining Cu-CCs was named m_2.

The formula to calculate the mass of Cu_2O/CuO is as below:

$$m\ (Cu_2O/CuO) = m_1 - m_2 \quad (1)$$

The total mass of the $Cu_2O/CuO@Cu$ composites on carbon cloth is as below:

$$m(Cu_2O/CuO@Cu)=m_1-m(\text{carbon cloth}) \quad (2)$$

The mass ratio of the Cu_2O/CuO and $Cu_2O/CuO@Cu$ are 2.85 mg cm^{-1} and 4.31 mg cm^{-1}, respectively

2 Supplementary Figures and Tables

Supplementary Table1 | The composition of the sensitizing solution.

Chemical	Reagents	Concentration
Stannous chloride	$SnCl_2 \cdot H_2O$	30 g L^{-1}
Hydrochloric acid	HCl	50 mL L^{-1}

Supplementary Table2 | The composition of the activating solution

Chemical	Reagents	Concentration
Silver nitrate	$AgNO_3$	2g L^{-1}
Ammonium hydroxide	$NH_3\ H_2O$	5mL L^{-1}

Supplementary Table3 | The composition of the electroless copper plating solution

**Chemical**	**Reagents**	**Concentration**
Copper sulfate	$CuSO_4 \cdot 5H_2O$	$14g \cdot L^{-1}$
Nickel chloride	$NiCl_2 \cdot 6H_2O$	$4\ g \cdot L^{-1}$
Formaldehyde	HCHO	$53\ ml \cdot L^{-1}$
Potassium sodium tartrate	$NaKC_4H_4O_6 \cdot 4H_2O$	$45.5\ g \cdot L^{-1}$
Sodium hydroxide	NaOH	$9\ g \cdot L^{-1}$
Sodium carbonate	Na_2CO_3	$4.2\ g \cdot L^{-1}$

Supplementary Table4 | Comparison of the electrochemical properties of the as-fabricated Cu_2O/CuO@Cu-CCs with previously reported Cu_2O or CuO based and other positive electrode

Material	Fabrication method	Current collector	Electrolyte	Specific capacitance	Rate performance	Reference
(CVO) Cu@CuO	cyclic voltammetry oxidization	Cu foam	6 M KOH	1.674 Fcm^{-2} (594.27 Fg^{-1})	2 $mAcm^{-2}$ (0.71 Ag^{-1})	(Liu et al., 2018)
CuO/CFF	Hydrothermal	Carbon cloth	6 M KOH	839.9 Fg^{-1}	1 mVs^{-1}	(Xu et al., 2016)
Co-doped Cu-MOF/$Cu_{2+1}O$	solvothermal method	Cu foam	6 M KOH	1.548 Fcm^{-2} (518.58Fg^{-1})	2 mA cm^{-2} (0.67 A g^{-1})	(Cao et al., 2019)
Cu_2O/MWCNs	the RTILs-assisted sputtering	Ni foam	6 M KOH	357 F g^{-1}	10 A g^{-1}	(Lu et al., 2018)
CuO/ZnO nanocomposits	Annealed in air	Ni foam	2 M KOH	579.5 F g^{-1}	5 A g^{-1}	(Lamberti et al., 2016)
Cu_2O/CuO nanocube	Hydrothermal	Cu foam	1M NaOH	400$mFcm^{-2}$	2 mA cm^{-2}	(Chen et al., 2017)
Cu_2O/CuO@Cu-CCs	solvothermal method	Carbon cloth	6 M KOH	1.71 Fcm^{-2} (835.2F g^{-1})	10 mA cm^{-2} （3.57 Ag^{-1}）	This work

3 Supplementary Figures

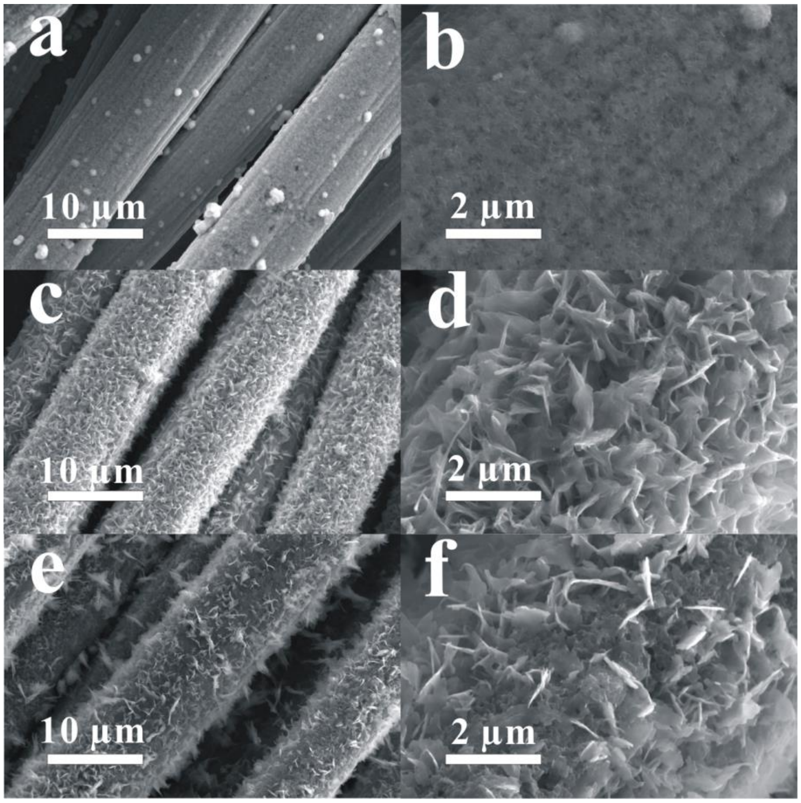

Supplementary Figure 1 | SEM images of Cu_2O/CuOnanosheets obtained from different eroding time. (a) 5 min, (c) 25 min,(e) 45min. (b), (d), (f) are the magnified images of (a), (c), (d), respectively.

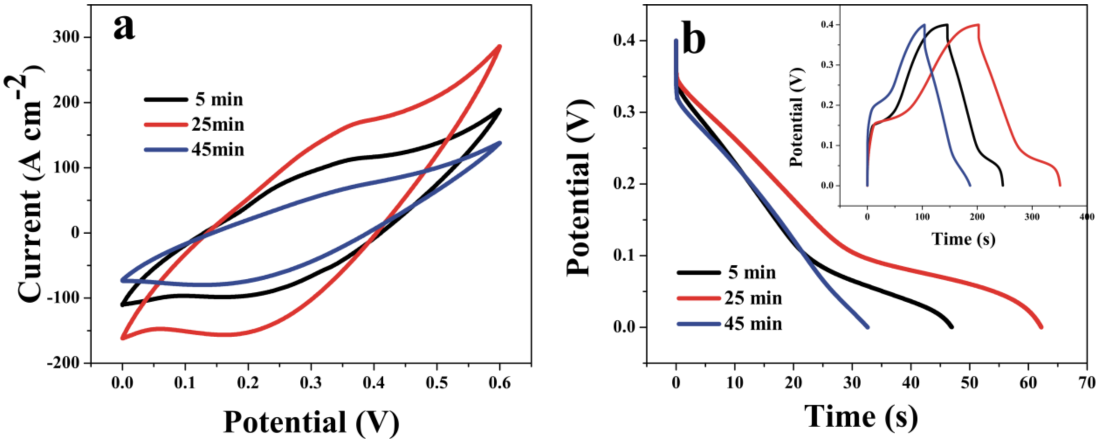

Supplementary Figure 2 | Electrochemical characterization of the Cu_2O/CuO@Cu-CCs eroding various time arrays electrode in 6 M KOH aqueous solution. (a) CV caves at a scan rate of 30 mV s^{-1}, (b) GCD caves under a current density of 10 mV cm^{-2}.

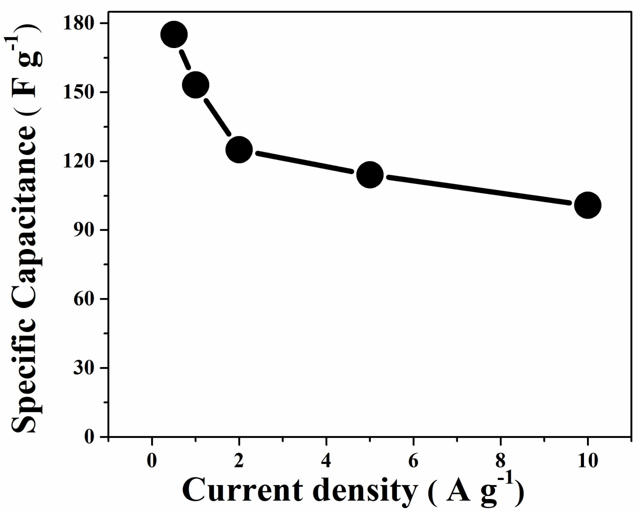

Supplementary Figure 3 | Specific capacitance of ASC devices under different current densities.

REFERENCES

Cao X., Cui L., Liu B., Liu Y., Jia D., Yang W. et al. (2019). Reverse synthesis of star anise-like cobalt doped Cu-MOF/$Cu_{2+1}O$ hybrid materials based on $Cu(OH)_2$ precursor for high performance supercapacitors. *J. Mater. Chem. A* 7, 3815-3827 .doi: 10.1039/C8TA11396C

Chen J. S., Huang S. P., Xu L. and Blackwood D. J. (2017). Sodium-Salt-Promoted Growth of Self-Supported Copper Oxides with Comparative Supercapacitive Properties. *ChemElectroChem* 4, 3188-3195. doi: 10.1002/celc.201700804.

Lamberti A., Fontana M., Bianco S. and Tresso E. (2016). Flexible solid-state Cu_xO-based pseudo-supercapacitor by thermal oxidation of copper foils. *Int. J. Hydrogen Energ.* 41, 11700-11708. doi: 10.1016/j.ijhydene.2015.12.198.

Liu Y., Cao X., Jiang D., Jia D. and Liu J. (2018). Hierarchical CuO nanorod arrays in situ generated on three-dimensional copper foam via cyclic voltammetry oxidization for high-performance supercapacitors. *J. Mater. Chem. A* 6. doi: 10.1039/c8ta00945g

Lu Y., Xu J. L., Ren S., Zhong Y. N., Gao X. and Wang S. D. (2018). Ionic-liquid-assisted one-pot synthesis of Cu_2O nanoparticles/multi-walled carbon nanotube nanocomposite for high-performance asymmetric supercapacitors. *Rsc Adv.* 8, 20182-20189. doi: 10.1039/C8RA02951B

Xu W., Dai S., Liu G., Xi Y., Hu C. and Wang X. (2016). CuO Nanoflowers growing on Carbon Fiber Fabric for Flexible High-Performance Supercapacitors. *Electrochim. Acta* 203, 1-8. doi: 10.1016/j.electacta.2016.03.170.
